# Tumor BRCA Testing in High Grade Serous Carcinoma: Mutation Rates and Optimal Tissue Requirements

**DOI:** 10.3390/cancers12113468

**Published:** 2020-11-21

**Authors:** Gulisa Turashvili, Conxi Lazaro, Shengjie Ying, George Charames, Andrew Wong, Krista Hamilton, Denise Yee, Evangeline Agro, Martin Chang, Aaron Pollett, Jordan Lerner-Ellis

**Affiliations:** 1 Department of Pathology and Laboratory Medicine, Mount Sinai Hospital and University of Toronto, Toronto, ON M5G 1X5, Canada; clazaro@iconcologia.net (C.L.); shengjie.ying@mail.utoronto.ca (S.Y.); George.Charames@sinaihealth.ca (G.C.); Andrew.Wong@sinaihealth.ca (A.W.); Krista.Hamilton@sinaihealth.ca (K.H.); Denise.Yee@sinaihealth.ca (D.Y.); Evangeline.Agro@sinaihealth.ca (E.A.); Martin.Chang@uvmhealth.org (M.C.); Aaron.Pollett@sinaihealth.ca (A.P.); Jordan.Lerner-Ellis@sinaihealth.ca (J.L.-E.); 2 Hereditary Cancer Program, Catalan Institute of Oncology, ONCOBELL-IDIBELL, 08908 Barcelona, Spain; 3 Cancer Network Consortium for Biomedical Research (CIBERONC), 08908 Barcelona, Spain; 4 Women’s College Research Institute, Women’s College Hospital, Toronto, ON M5S 1B2, Canada

**Keywords:** high-grade serous carcinoma, BRCA, tumor sequencing, PARP inhibitors

## Abstract

**Simple Summary:**

Approximately 25% of women diagnosed with tubo-ovarian high-grade serous carcinoma have germline deleterious mutations in *BRCA1* or *BRCA2*, characteristic of hereditary breast and ovarian cancer syndrome, while somatic mutations have been detected in 3–7%. We set out to determine the mutation rates and optimal tissue requirements for tumor *BRCA* testing in 291 tissue samples. Initial testing was successful in 78% and deemed indeterminate in 13%. Repeat testing was successful in 67% of retested samples, with an overall success rate of 86%. Clinically significant *BRCA* variants were identified in 17% of patients. Successful sequencing was dependent on sample type, tumor cellularity and size (*p* ≤ 0.001) but not on neoadjuvant chemotherapy or age of blocks. In summary, our study shows a 17% tumor *BRCA* mutation rate, with an overall success rate of 86%. Biopsy and cytology samples and post-chemotherapy specimens can be used, and optimal tumors measure ≥5 mm in size with at least 20% cellularity.

**Abstract:**

Background: Approximately 25% of women diagnosed with tubo-ovarian high-grade serous carcinoma have germline deleterious mutations in *BRCA1* or *BRCA2*, characteristic of hereditary breast and ovarian cancer syndrome, while somatic mutations have been detected in 3–7%. We set out to determine the BRCA mutation rates and optimal tissue requirements for tumor BRCA testing in patients diagnosed with tubo-ovarian high-grade serous carcinoma. Methods: Sequencing was performed using a multiplexed polymerase chain reaction-based approach on 291 tissue samples, with a minimum sequencing depth of 500X and an allele frequency of >5%. Results: There were 253 surgical samples (87%), 35 biopsies (12%) and 3 cytology cell blocks (1%). The initial failure rate was 9% (25/291), including 9 cases (3%) with insufficient tumor, and 16 (6%) with non-amplifiable DNA. Sequencing was successful in 78% (228/291) and deemed indeterminate due to failed exons or variants below the limit of detection in 13% (38/291). Repeat testing was successful in 67% (28/42) of retested samples, with an overall success rate of 86% (251/291). Clinically significant (pathogenic, likely pathogenic) variants were identified in 17% (48/276) of complete and indeterminate cases. Successful sequencing was dependent on sample type, tumor cellularity and size (*p* ≤ 0.001) but not on neoadjuvant chemotherapy or age of blocks (*p* > 0.05). Conclusions: Our study shows a 17% tumor BRCA mutation rate, with an overall success rate of 86%. Biopsy and cytology samples and post-chemotherapy specimens can be used for tumor *BRCA* testing, and optimal tumors measure ≥5 mm in size with at least 20% cellularity.

## 1. Introduction

Ovarian cancer is the 7th most common malignancy in women worldwide [1]. Epithelial tumors account for approximately 90% of all ovarian cancers and are comprised of five distinct disease groups, with different clinical presentations, pathogenesis, chemosensitivity and prognosis [2]. High-grade serous carcinoma (HGSC) is the most common histological subtype of epithelial ovarian cancer accounting for 70% of cases and majority of deaths, with a median overall survival of 41 months [1,3]. Up to 25% of women diagnosed with HGSC have germline deleterious mutations in *BRCA1* or *BRCA2* characteristic of hereditary breast and ovarian cancer syndrome (HBOC) [4,5,6], while somatic mutations have been detected in 3–7% [7,8,9,10,11,12]. Of all patients with tumor *BRCA1/2* variants, 54–74% are germline and 27–46% are somatic, i.e., present only in the tumor tissue [10,12,13,14].

The *BRCA1* (chromosome 17q21) and *BRCA2* (chromosome 13q12.3) are tumor suppressor genes that encode for proteins essential in DNA double strand break repair by homologous recombination (HR) [15,16,17]. In women with HBOC, cells with a “second-hit” leading to HR deficiency rely on alternative error-prone mechanisms of DNA repair that leads to an increased risk for the development of a variety of malignancies involving the breast as well as ovary, fallopian tube or peritoneum [18,19]. The presence of *BRCA* mutation in HGSC has important prognostic and predictive implications. Tumor cells with HR deficiency have been shown to display greater sensitivity to platinum-based chemotherapy regimens in both breast and ovarian cancer patients [20,21,22]. In addition, HR deficient tumors can also be treated with novel poly adenosine diphosphate ribose polymerase (PARP) inhibitors. PARP enzymes are important in DNA single-strand break repair. PARP inhibitors selectively target HR-deficient cancer cells and lead to cell death through the mechanism of synthetic lethality [23,24,25,26]. PARP inhibitors have been demonstrated to extend progression free survival in HGSC patients with the greatest effectiveness in patients with *BRCA1/2* mutations [27,28,29,30,31].

The first PARP inhibitor Olaparib was approved by the United States Food and Drug Administration (FDA) in 2016 as a maintenance treatment for *BRCA*-mutated recurrent HGSC following ≥3 lines of chemotherapy, and extended to treatment following first-line chemotherapy in 2018. Tumor *BRCA* testing and PARP inhibitor monotherapy for the maintenance treatment of adult patients with *BRCA*-mutated platinum-sensitive relapsed (PSR) HGSC was first approved by Health Canada in April 2016. Currently, tumor *BRCA* testing is performed reflexively for newly diagnosed HGSC patients in Ontario in several referral centres. Although formalin-fixed paraffin-embedded (FFPE) samples can be used for molecular studies, there is a wide variety of pre-analytical and analytical variables that can affect the performance of molecular assays, including cold ischemic time, length of fixation, storage conditions, age of paraffin blocks etc. [32,33,34]. The use of FFPE tumor tissue for *BRCA* variant analysis using next-generation sequencing (NGS) as well as the feasibility of the implementation for such tests in clinical practice have been investigated previously [7,35]. Tumor testing for *BRCA* variant screening has been suggested to be more efficient in selecting HGSC patients for genetic counseling as well as for PARP inhibitor therapy [13]. A combination of NGS and copy number variant multiplex ligation-dependent probe amplification has been reported to have a sensitivity of 98% in the training cohort of 50 patients, and 100% in the prospective cohort of 66 patients [13]. Another prospective analysis of FFPE samples from 223 patients with epithelial ovarian cancer showed that the tumor *BRCA* testing using NGS had a success rate of 99.1% (221/223), with a 28.1% rate of pathogenic/likely pathogenic mutations and 87% concordance rate between germline and tumor *BRCA* tests [36].

In this study, we present the results of tumor *BRCA1/2* testing in a cohort of 291 HGSC patients performed in the clinical setting at a tertiary hospital. We report the rate of tumor *BRCA1/2* mutations, optimal tissue requirements and challenges encountered such as variable quality of tissue samples and success rates following repeat testing.

## 2. Results

### 2.1. Overview of Clinical Data

FFPE samples from a total of 291 patients diagnosed between 2004 and 2019 were received for tumor *BRCA1* and *BRCA2* analysis during the study period. The median patient age was 65 years (range 34–89). Most (87%, 253/291) samples were from surgical excisions, with 12% (35/291) biopsies and 1% (3/291) cytology cell blocks from peritoneal fluid. Of tissue samples, most cases (41.2%, 120/291) were ovarian, 11% (32/291) were tubal and “adnexal” each, 20% (58/291) were omental, although other tissues were also represented (16.8%, 49/291). Most patients (70.8%, 206/291) were chemotherapy naïve (Table 1).

### 2.2. Initial Testing

**Figure 1:**
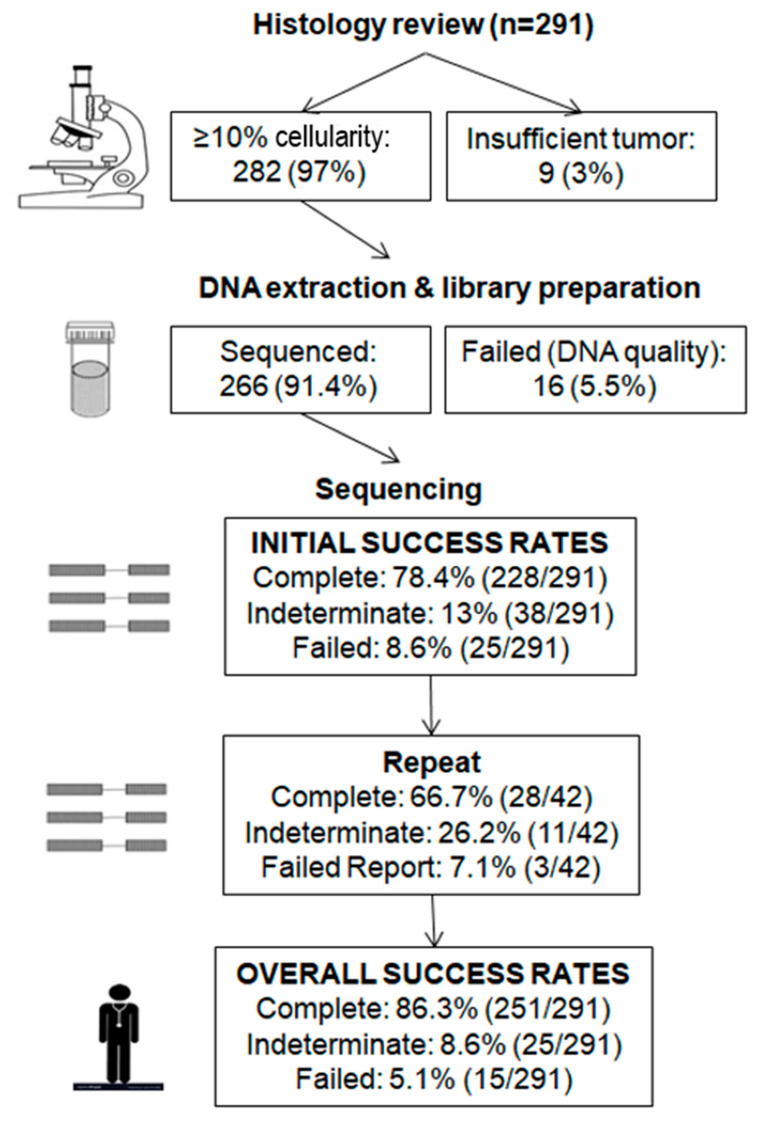
Schematic summary of success rates of tumor *BRCA* analysis. Complete report: all exons of *BRCA1* and *BRCA2* sequenced; indeterminate report: ≥1 exon of *BRCA1* and/or *BRCA2* with low coverage depth or pathogenic or likely pathogenic variants below the limit of detection; failed report: unsuccessful *BRCA* analysis.

Tumor cellularity was analyzed by a single pathologist prior to sequencing. Samples with a cellularity of <10%, irrespective of tumor size, were not eligible for sequencing under the assumption that a heterozygous variant would not meet the 5% limit of detection for this test. Of 291 samples, 9 (3%) had insufficient tumor with <10% cellularity and thus were not submitted for DNA extraction, 16 (5.5%) had suboptimal DNA quality and/or quantity and thus were not submitted for sequencing. Overall, the initial failure rate was 8.6% (25/291) (Figure 1). Of 266 (91.4%) samples that were sequenced, complete reports were generated in most cases (78.4%, 228/291), while 13% (38/291) was considered indeterminate. Indeterminate reports included those with either failed exons due to the possibility of uncaptured pathogenic or likely pathogenic variants within the failed exons or with pathogenic or likely pathogenic variants below the limit of detection (LOD) due to uncertainty over true presence of clinically significant variants below LOD.

### 2.3. Repeat Testing

**Figure 2:**
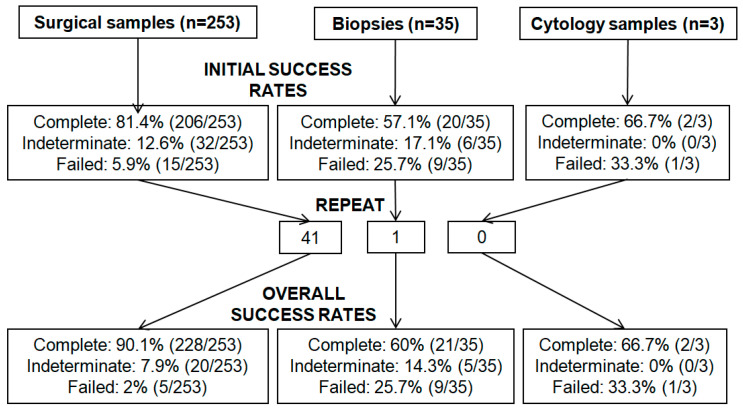
Success rates of tumor *BRCA* analysis by specimen type. Complete report: all exons of *BRCA1* and *BRCA2* sequenced; indeterminate report: ≥1 exon of *BRCA1* and/or *BRCA2* with low coverage depth or pathogenic or likely pathogenic variants below the limit of detection; failed report: unsuccessful *BRCA* analysis.

Repeat testing was performed in 42 cases, including all 16 failed (insufficient and/or non-amplifiable DNA) samples and 26 of 38 indeterminate (19 with low coverage depth, 11 with variants below the LOD, 1 with a large number of variants of uncertain significance (VUS)) samples (Figure 2). Successful results were obtained in 66.7% (28/42) of cases, 7.1% (3/42) failed, while the remaining 26.2% (11/42) were still indeterminate. New paraffin blocks were tested in three patients, of which one yielded a complete report. Repeat testing identified a pathogenic variant in three failed cases and four cases with low depth of coverage. Of interest, one sample had low coverage depth in exons 22–23 in *BRCA1* in both initial and repeat tests, which was confirmed using a second independent methodology (following enrichment using the Illumina TruSight Rapid Capture Kit) [37] to be a likely pathogenic deletion of exons 22–23.

Thus, overall success rates based on the 291 samples were as follows: complete reports issued in most cases (86.3%, 251/291), failed reports in 5.1% (15/291), while 8.6% (25/291) of cases were still considered indeterminate (Figure 2). Of these 25 patients, 15 had <5 exons with low coverage, 6 had 5–9 exons with low coverage, and 4 had ≥10 exons with low coverage; the remaining exons were successfully sequenced.

### 2.4. Predictors of Successful Tumor BRCA Analysis

We next assessed how sample type, tumor cellularity, tumor size, neoadjuvant chemotherapy or age of paraffin blocks affected the rates of successful tumor *BRCA* analysis. Detailed analysis of failure rates based on tumor cellularity and tumor size showed higher rates of failed reports among samples with lower tumor cellularity and smaller tumors. More specifically, failure rates were 4% (9/226) in samples with ≥50% cellularity, 15.2% (7/46) in samples with cellularity >10% but <50%, and 47.4% (9/19) in samples with ≤10% cellularity (*p* < 0.0001). When rates of successful tumor *BRCA* analysis were analyzed by tumor size, failure rates were 6.6% (17/259) in samples measuring ≥10 mm, 7.1% (1/14) in samples measuring 5–9 mm, and 38.9% (7/18) in samples measuring ≤4 mm (*p* < 0.0001). Comparison of specimen types demonstrated a failure rate of 25.7% (9/35) in biopsies, 33.3% (1/3) in cytology specimens and 5.9% (15/253) in surgical samples (*p* = 0.001). There was no association with post-neoadjuvant chemotherapy, age of the paraffin blocks tested or referring site (*p* ≥ 0.396) (Table 2). The mutation rate was 13% (19/144) in patients diagnosed before 2018 versus 20% (29/147) for patients diagnosed after 2018 (Fisher`s *p* = 0.16).

### 2.5. Histologic Analysis

We retrospectively assessed histologic variables previously reported to be predictive of *BRCA1/2* status in surgical specimens from 172 chemotherapy naïve patients. These variables included the Solid, pseudo-Endometrioid and Transitional cell-like (SET) features, necrosis, grade 3 nuclei, abundant tumor-infiltrating lymphocytes (TILs) and metastases with pushing or micropapillary growth patterns. Formal statistical analysis was underpowered due to the small number of *BRCA1* and *BRCA2* mutant samples; however, histologic features in tissue samples with and without *BRCA* mutations are summarized in Table 3.

### 2.6. Clinically Significant BRCA Mutations

Pathogenic or likely pathogenic variants were identified in 17.4% (48/276) of complete and indeterminate cases, including *BRCA1* variants in 30 patients and *BRCA2* variants in 18 patients (Table 4 and Table S1). The most common type of mutations was substitutions (23/48, 47.9%). Of these, 12 created a premature stop codon, 7 were splicing alterations, and 4 were missense mutations. The remaining mutations were small deletions (39.6%, 19/48), small duplications (6.2%, 3/48), combined indels (4.2%, 2/48), and small insertions (2.1%, 1/48), all of which resulted in a frameshift (Table 4). Most patients with mutations (60.4%, 29/48) were 60 years or older, including 16 patients aged 70–79 and 13 patients aged 60–69. Only eight patients (16.7%) were 40–49 years of age.

In addition, our study identified 33 variants of uncertain significance (VUS) in 27 patients, including 23 patients with 1 VUS, 3 patients with 2 VUS, and 1 patient with 4 VUS. Five of the 23 patients with 1 VUS also had a pathogenic variant. Almost all VUS were substitution variants (97%, 32/33), including 26 missense variants, 3 silent variants, 2 splicing alterations, 1 variant located in the 5’ untranslated region, and 1 in-frame deletion. Unlike pathogenic variants, VUS were more common in *BRCA2* (69.7%, 23/33) than *BRCA1* (33.3%, 11/33) (Table S2).

### 2.7. Loss of heterozygosity (LOH) Analysis

This molecular test was evaluated for copy number variant detection by way of assessing allelic imbalances in tumors for individual variants, although amplicon sequencing may not be the best approach for the detection of copy number alterations or LOH. Tumors were considered LOH positive if there was an allelic imbalance of two or more variants in the same gene with allele frequencies between 5 and 45% or 55 and 95%, regardless of the presence of a pathogenic variant. Among the 276 patients with complete or indeterminate *BRCA1/2* sequencing results, 204 (73.9%) had LOH in either *BRCA1* only (59, 29%), *BRCA2* only (78, 38.2%), or both *BRCA1* and *BRCA2* (67, 32.8%). By this definition, 39 of 48 (81.3%) subjects with a pathogenic variant had LOH. Among these, 31 (79.5%) tumors showed LOH in the same gene as the mutation with 14 (35.9%) in *BRCA1* and 17 (43.6%) in *BRCA2*; 8 (20.5%) samples had LOH in the non-mutated *BRCA* gene (Table S2). By applying this method of determining LOH, we assessed whether or not the LOH occurred at the same allele as the pathogenic variant in order to investigate bi-allelic inactivation. Among the 48 pathogenic variants, 29 (60.4%) had an allele frequency greater than 0.55. Of these, 11 (37.9%) had another variant with an allele frequency below 0.45; 10 (34.5%) had another variant with an allele frequency above 0.55, but no variant below 0.45; 8 (27.6%) did not have any other variants in the same gene and did not meet LOH criteria defined here as allelic imbalance in at least two variants.

## 3. Discussion

Our cohort consists of 291 HGSC patients who underwent tumor *BRCA1/2* testing between 1 September 2018 and 31 May 2019 in our laboratory to determine eligibility for PARP inhibitor therapy. Tumor *BRCA1/2* variant status was determined for 276 patients (251 complete reports with fully sequenced *BRCA1* and *BRCA2* genes, 25 indeterminate reports with at least one failed exon), of which 17.4% (48/276) had a pathogenic/likely pathogenic variant and 8.3% (23/276) carried a VUS. The overall mutation rate of 17.4% in our study may be on the lower end of the spectrum but appears comparable to the combined germline and somatic mutation rate of 16.7–28% reported in previous studies [7,10,12,14,35]. We hypothesize that this is primarily due to ascertainment bias as some patients diagnosed before 2018 may have already been tested negative for a germline *BRCA* mutation. In support of our hypothesis, we found that the pathogenic/likely pathogenic mutation rate for patients diagnosed before 2018 was 13%, whereas the diagnostic yield was 20% after 2018. Although not statistically significant, the post-2018 rate is more consistent with what we had previously observed in the hereditary breast and ovarian cancer germline genetic testing program in Ontario [38] as well as with the existing literature [7,10,12,35]. However, given that the molecular test does not distinguish between somatic or germline mutations, we were not able to assess the actual somatic mutation rate in this cohort by way of ruling out germline variation.

The FFPE tumor samples were first assessed by analyzing tumor cellularity and tumor size. In general, we observed that samples with higher tumor cellularity and larger tumors had better outcomes of tumor *BRCA* analysis, with failure rates at 4% in samples with ≥50% cellularity versus 47.4% in samples with ≤10% cellularity, and 6.6% in samples measuring ≥10 mm versus 38.9% in samples measuring ≤4 mm (*p* < 0.0001). In addition, surgical specimens had better analysis yield compared to biopsies and cytology specimens with failures rates of 5.9%, 25.7% and 33.3%, respectively (*p* = 0.001), although only three cytology samples were included in the study. Interestingly, neoadjuvant chemotherapy or age of paraffin blocks did not affect the failure rate. These results support the use of FFPE samples with high tumor cellularity. In addition, samples measuring < 5 mm in size have an almost 40% probability of failure, highlighting the importance of recording tumor size in addition to cellularity. It is also worth noting that despite this association between low tumor cellularity and small tumor size with higher failure rates, samples with 10% tumor cellularity (the lowest eligible cellularity) and those measuring ≤4 mm were still successfully sequenced. This indicates that tumors measuring ≤4 mm or tumors with 10% cellularity may still yield sufficient DNA for sequencing, although a higher proportion of failures would be expected. Thus, sequencing may be attempted on these samples if and when more optimal specimens are not available.

In order to maximize diagnostic yield, a second sample, if available, was sequenced if the first test failed or resulted in an indeterminate report with variants below the LOD or low coverage depth in at least one exon. A report with low coverage depth was considered indeterminate because there may be pathogenic variants within the exons of low coverage that were not detected. Of the 291 patients, 8.6% had failed reports, and 13% had indeterminate reports following the first test per patient. Among 42 patients that had a second sample tested, complete reports were successfully generated in 66.7%. A pathogenic variant was also identified in seven patients (three failed, four with low depth coverage), demonstrating high value in repeating previously failed samples as well as samples with low depth coverage. Only three repeats were done on a different tissue block, suggesting that a new block may not be necessary. In summary, after repeat testing, the proportion of conclusive reports increased from 78.4% to 86.3%, while the number of indeterminate reports decreased from 13% to 8.6%, demonstrating that repeat testing is an effective method of increasing diagnostic yield in a clinical setting for challenging tissues such as FFPE.

With the incorporation of *BRCA1/2* sequencing into HGSC treatment, it is of interest to associate histologic characteristics with *BRCA1/2* mutation status. Various histologic features have been reported to be predictive of *BRCA* mutations including the presence of SET features, necrosis, grade 3 nuclei, tumor infiltrating lymphocytes and mitotic index as well as metastases with pushing or “medullary-like” invasion or infiltrative invasion composed exclusively of micropapillae [39,40,41]. Although the histologic criteria developed in previous studies have limited positive predictive value, they have been more effective at predicting the absence of *BRCA1*/2 mutations with negative predictive value of >95% [39,40]. Our study was underpowered for formal statistical analysis. However, the aforementioned histologic features could be another tool used by clinicians to prioritize accessibility to *BRCA1/2* testing in situations where this is necessary.

LOH was not included in most clinical trials to determine if individuals with this type of genomic alteration might be responsive to PARP inhibitors [27,31,42,43,44], as LOH status is not considered to be the type of pathogenic variant that would be eligible for PARP inhibitors. We assessed LOH status for every patient based on allelic imbalances; however, it was not used for clinical decisions. In our study, 73.9% of patients had LOH which could be due to high genomic instability of HGSC. Some of the variants had low (<10%) allele frequency even in tumors with >80% cellularity and LOH. This could be explained as artifacts or low level somatic variation. Alternatively, if a non-tumor variant occurred in a sample with 80% cellularity as a heterozygote, the theoretical allelic fraction would be 10%.

Previous studies have suggested that HGSC with retention of the normal *BRCA1* or *BRCA2* allele (absence of locus-specific LOH) may have lower HR deficiency scores compared with tumors with locus-specific LOH, and the latter may be used to predict primary resistance to PARP inhibitors in *BRCA* mutation carriers [45]. Although LOH was not investigated in clinical trials for PARP inhibitor therapy, *BRCA* wild-type patients with LOH were analyzed as a subgroup in the ARIEL 3 clinical trial for rucaparib and demonstrated improved progression free survival compared to placebo [46]. If LOH alone is sufficient for PARP inhibitors to be effective, treatment with PARP inhibitors could potentially be extended to 73.9% of patients in our study who tested positive for LOH based on our criteria. In theory, however, the effective mechanism of PARP inhibitors requires inactivation of both copies of either *BRCA1* or *BRCA2*. This is supported by in vitro and in vivo studies that demonstrate mutated cells in the heterozygous state are significantly less responsive to PARP inhibitors compared to homozygous mutants [47,48,49,50,51]. Based on this theory, only patients with bi-allelic inactivation of *BRCA1/2* should be eligible, which would exclude the currently eligible hypothetical subset of patients who are heterozygous for a *BRCA1/2* mutation with no apparent inactivation of the second copy. We found that among the 48 patients with a *BRCA* mutation, 39 met our general criteria for LOH, but of these only 21 (43%) had a pathogenic variant allele frequency above 0.55 that supported bi-allelic inactivation. These rates are considerably lower than previously reported rates of 84–100% of LOH in germline mutant *BRCA1*/2 [45,52]. Two mutations had an allele frequency between 0.45 and 0.55 while the remaining 16 had an allele frequency below 0.45. Some of these low frequency mutations may indicate mutations that were somatic in origin. Because LOH status was determined by a shift in allele frequency to 55–95%, we could not determine if it was caused by deletion, mitotic recombination or gene conversion, or other more complex chromosomal alterations which could result in copy number abnormality or copy neutral LOH. We also did not assess other potential methods of gene inactivation such as promoter methylation which would be a valuable future addition to this test. Additionally, it is generally more difficult to detect large deletions in DNA extracted from FFPE tissue compared to germline DNA [32].

Given that tumor *BRCA1/2* testing identifies both germline and somatic mutations, we endorse universal tumor testing in newly diagnosed HGSC patients. The universal tumor *BRCA1/2* testing workflow has been shown to be a feasible, effective and robust option in daily pathology practice, and well perceived by gynecologists and patients [14]. It maximizes mutation detection rate (at least 16.7% versus 9.5% with universal genetic predisposition testing) and effectively identifies patients who are eligible for PARP inhibitor therapy. In addition, it may also serve as a screening tool to tailor genetic counseling and may improve uptake of genetic predisposition testing in HGSC patients [14]. It should also be noted that there are other genes in the HR family, such as *ARID1A*, *ATM*, *ATRX*, *BAP1*, *BARD1*, *BLM*, *BRIP1*, *CHEK1/2*, *PALB2*, *RAD50*, *RAD51*, *RAD51B*, *EMSY*, that may be recognized as eligibility criteria for PARP inhibitor therapy in the future [53,54,55]. Lastly, PARP inhibitors are increasingly being used for patients with platinum-sensitive HGSC regardless of *BRCA* status [56,57].

Our study has several limitations: (a) germline mutation status of individuals whose tumor tissues tested positive for a *BRCA1/2* mutation was unknown; thus, it is unclear if the mutations detected were of germline or somatic origin (albeit this does not affect eligibility for PARP inhibitors); (b) many cases were referred with only one submitted paraffin block available for histologic review, introducing a selection bias and limiting our assessment of *BRCA* mutation associated morphologic features.

## 4. Materials and Methods 

### 4.1. Study Population and Reference Laboratory

A total of 291 patients with HGSC underwent tumor *BRCA* testing in the Advanced Molecular Diagnostics laboratory at Mount Sinai Hospital between October 2018 and May 2019. Patient eligibility for tumor *BRCA* testing was determined based on the Ontario Ministry of Health and Long-Term Care and Cancer Care Ontario guidelines. This included patients diagnosed with HGSC of the ovarian, fallopian tube or primary peritoneal origin, whose *BRCA* mutation status was either unknown or potentially negative for germline testing at the time of testing. Patients who have previously tested positive for a germline *BRCA* mutation were not eligible. Samples were received from 15 different hospitals including Sinai Health System. Germline *BRCA* status was not provided by referring clinicians. The study was approved by the Research Ethics Board (19-0071E).

The Advanced Molecular Diagnostics laboratory at Mount Sinai Hospital is accredited by the Institute of Quality Management in Healthcare (IQMH) to ISO 15189:2012/15190:2003. All tests were validated according to accepted practice guidelines for molecular genetic testing of the American College of Medical Genetics (ACMG), the College of American Pathologists (CAP), and the Clinical and Laboratory Standards Institute (CLSI).

### 4.2. Tissue Samples and DNA Extraction

Microscopic slides were reviewed to confirm the diagnosis of HGSC, determine tumor cellularity in increments of 10 and maximum linear dimension in millimeters, and circle tumor tissue. Slides from chemotherapy naïve patients were also reviewed by a single pathologist blinded to sequencing results (GT) for specific histologic features associated with *BRCA* mutations. Tumors were then macrodissected from six 10-µm FFPE tissue sections with a minimum tumor cellularity of 10%. DNA was extracted from macrodissected tumor samples using the Qiagen QIAsymphony DSP DNA Mini Kit on the Qiagen QiaSymphony SP Automated DNA Extractor (Qiagen, Venlo, The Netherlands). The quantity and quality of the DNA was determined by fluorescent spectrophotometric analysis using Qubit (Thermofisher, Waltham, MA, USA).

### 4.3. Library Preparation and Analysis

The Illumina AmpliSeq Library PLUS for the *BRCA* Panel kit (Illumina, SanDiego, CA, USA) was used to prepare multiplex PCR libraries for DNA sequencing according to the manufacturer instructions. The minimum acceptable tumor cellularity was 10%, while the minimum acceptable sample DNA Qubit concentration was >0.01 ng/µL in order to proceed with library preparation. Analysis included all exonic regions and flanking intronic sequences (±15 base pairs from the exon boundaries) of *BRCA1* and *BRCA2* genes. Amplicon coverage included two primer pools (Pool 1: 132 amplicons, Pool 2: 133 amplicons) that overlap all the exonic and flanking intronic regions of the *BRCA1* and *BRCA2* genes. After amplification, leftover primer sequences were digested and sequencing adapters were ligated to the amplicons. Libraries were amplified again, quantitated, normalized and pooled together for sequencing. A Qubit value of 0.37 ng/µL was required to achieve the normalized concentration (2 nM) for sample pooling to avoid preferential sequence amplification. Twelve tumor samples per library were pooled and run on an Illumina MiSeq instrument using a v2 Micro cartridge. Paired end reads 150 bp in each direction was used for this test. For repeat testing, DNA was re-extracted from the same block or a new block.

Quality control measures were followed per the provincial laboratory accreditation standards for DNA transfer (to dilution tubes or plate) and the plates had two unique identifiers to label each sample. In addition, proficiency testing is performed several times per year both internally and through external programs to assess performance and quality of the test.

### 4.4. Bioinformatic Analysis

Variant calling was performed using the DNA Amplicon Analysis Module V1.1.0 from Illumina with a minimum per base coverage of 500 reads. Quality metrics for each run were evaluated and needed to meet minimum acceptable threshold values based on prior clinically validated testing metrics. These are summarized as averages as follows: total Yield (G) of 1.81 (SD 0.1), number of reads passing filter (Reads PF) (M) of 5.75 (SD 0.32), Cluster Density (k/mm^2^) of 1153 (SD 89.85), Clusters Passing Filter of 91.9% (SD 2.78) and >Q30 of 94.4% (SD 0.8) (Table S3).

A minimum per base read depth 500X was required to pass filter; the LOD (limit of detection) was determined to be 5% and a minimum of 5% frequency of the alternative allele was required for reporting.

The analytical sensitivity was > 99% and specificity was 100% for DNA substitutions and small deletions or duplications (up to 5 bp) as well as exon-level or full gene deletions or duplications, as demonstrated during the validation process in our laboratory before using the test in the clinical setting. This test does not reliably detect chromosomal aberrations or rearrangements.

To identify deletions or loss of heterozygosity (LOH), a comprehensive analysis of all variants in both *BRCA* genes was used based on internal validation metrics. Ranges of variant frequencies for LOH were as follows: no evidence of LOH (45–55%), strong evidence of LOH (5–45%) (55–95%), and not informative evidence of LOH (0–5%) (95–100%). LOH was reported if 2 or more variants from the same gene had strong evidence of LOH (Tables S4,S5, Figure S1).

### 4.5. Reporting and Variant Interpretation

**Figure 3:**
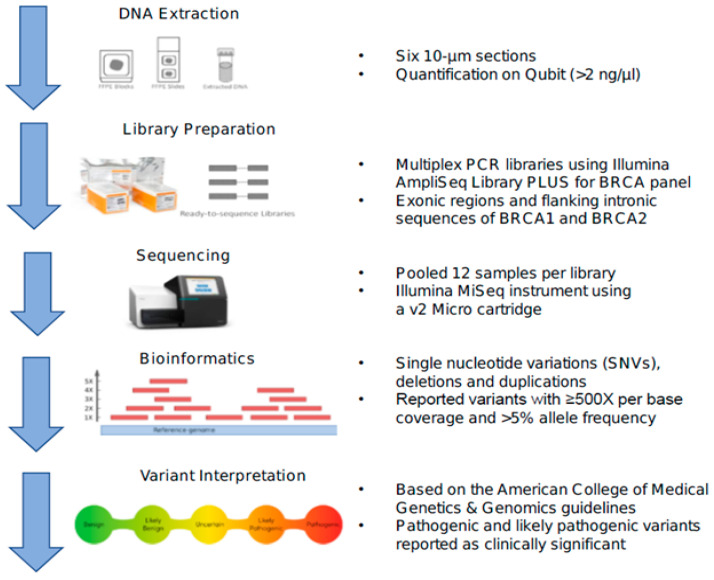
Schematic summary of laboratory workflow and analysis.

DNA variants were described using HGVS (Human Genome Variation Society) nomenclature and variant interpretation and classification was based on the American College of Medical Genetics and Genomics (ACMG) 2015 guidelines [58]. Pathogenic and likely pathogenic variants were reported as clinically significant. Variants of uncertain significance were also included in the report, while benign and likely benign variants were not included (workflow scheme in Figure 3). The report emphasized that the results were specific to tumor tissue and would not decipher between germline versus somatic mutations. Thus, genetic counselling and germline testing was recommended by the laboratory for individuals with a pathogenic or likely pathogenic variant.

If DNA extraction, library preparation or sequencing failed (defined as one or more exons falling below the target threshold coverage of 500X), a report was generated with a statement recommending repeat testing. If a pathogenic or likely pathogenic variant was identified, repeat testing was not recommended regardless of whether or not other exons met the target threshold coverage. Additionally, in samples with pathogenic or likely pathogenic variants that were below the LOD (5%), an alternative sample was requested for confirmation testing. A maximum of two samples were tested for each patient.

### 4.6. Statistical Analysis

The significance of associations between variables was analyzed by using the chi-square test and Fisher’s two-sided exact test. The software used was SPSS 25.0 for Windows. Probability values of <0.05 were considered significant.

## 5. Conclusions

This prospective analysis demonstrates that our *BRCA*-tumor testing workflow is effective in identifying individuals who may benefit from PARP inhibitor treatment, with success rates ranging from 78.4% on initial testing to 86.3% following repeat testing. Our study shows a 17.4% tumor *BRCA* mutation rate, slightly lower compared to the literature most likely due to ascertainment bias of the studied population. Biopsy and cytology samples and post-chemotherapy specimens can be used, and optimal tumors measure ≥5 mm with at least greater than 10% cellularity. Variable quality of FFPE tissue remains a challenge but this was substantially alleviated through repeat testing with a 66.7% success rate. We also assessed LOH that may become a valuable diagnostic tool in the future if it is found to be clinically relevant in the context of PARP inhibitor therapy.

## Figures and Tables

**Table 1:** Description of the tissue samples analyzed.

Tissue Type	No (%)
Ovary	120 (41.2)
Fallopian tube	32 (11.0)
Adnexa	32 (11.0)
Omentum	58 (20.0)
Other	49 (16.8)
Post neoadjuvant chemotherapy	
Yes	85 (29.2)
No	206 (70.8)

**Table 2:** Summary of initial success rates of *BRCA1/2* sequencing based on clinical–pathologic variables.

Clinical–Pathologic Variables		Complete	Failed	Indeterminate	*Total*	*p*-Value (Pearson Chi-Square)
Sample type	Surgical	206 (81.4%)	15 (5.9%)	32 (12.7%)	253 (87%)	0.001
	Biopsy	20 (57.1%)	9 (25.7%)	6 (17.2%)	35 (12%)	
	Cytology	2 (66.7%)	1 (33.3%)	0	3 (1%)	
Tumor cellularity	≥50%	185 (81.9%)	9 (4.0%)	32 (14.1%)	226 (77.7%)	<0.0001
	>10% to <50%	34 (73.9%)	7 (15.2%)	5 (10.9%)	46 (15.8%)	
	≤10%	9 (47.4%)	9 (47.4%) *	1 (5.2%)	19 (6.5%)	
Tumor size	≥10 mm	206 (79.5%)	17 (6.6%)	36 (13.9%)	259 (89%)	<0.0001
	5–9 mm	13 (92.9%)	1 (7.1%)	0	14 (4.8%)	
	≤4 mm	9 (50.0%)	7 (38.9%)	2 (11.1%)	18 (6.2%)	
Chemotherapy	Yes	64 (75.3%)	9 (10.6%)	12 (14.1%)	85 (29.2%)	0.669
	No	164 (79.6%)	16 (7.8%)	26 (12.6%)	206 (70.8%)	
Age of tissue block, years	<3	111 (75.5%)	13 (8.8%)	23 (15.7%)	147 (50.5%)	0.396
	≥3	117 (81.3%)	12 (8.3%)	15 (10.4%)	144 (49.5%)	
Total		228 (78.4%)	25 (8.6%)	38 (13.0%)	291 (100%)	

* 9 samples with insufficient tumor.

**Table 3:** Summary of histologic features associated with *BRCA1/2* mutations in surgical specimens from a cohort of 172 chemotherapy-naïve patients.

Histologic Features	Mutation Total	*BRCA1* Mutation	*BRCA2* Mutation	No Mutation	Total
SET features					
S	13 (38.2%)	9 (45%)	4 (28.6%)	35 (25.4%)	48 (27.9%)
E	11 (32.4%)	7 (35%)	4 (28.6%)	21 (15.2%)	32 (18.6%)
T	4 (11.8%)	0	4 (28.6%)	2 (1.4%)	6 (3.5%)
All 3 features	2 (5.9%)	0	2 (14.3%)	2 (1.4%)	4 (2.3%)
Any 2 of 3 features	7 (20.6%)	7 (35%)	0	19 (13.8%)	26 (15.1%)
Any 1 of 3 features	2 (5.9%)	2 (10%)	0	14 (10.1%)	16 (9.3%)
Necrosis	27 (79.4%)	18 (90%)	9 (64.3%)	93 (67.4%)	120 (69.8%)
Grade 3 nuclei	15 (44.1%)	11 (55%)	4 (28.6%)	79 (57.2%)	94 (54.6%)
TILs	5 (14.7%)	2 (10%)	3 (21.4%)	14 (10.1%)	19 (11%)
Metastasis with pushing or micropapillary pattern	3 (8.8%)	2 (10%)	1 (7.1%)	4 (2.9%)	7 (4.1%)
Total	34 (19.8%)	20 (11.6%)	14 (8.2%)	138 (80.2%)	172 (100%)

SET, Solid, pseudo-Endometrioid and Transitional cell-like; TILs, tumor-infiltrating lymphocytes.

**Table 4:** Summary of pathogenic and likely pathogenic *BRCA* variants identified.

No	Exon	Variant	Allele Frequency	Cellularity, %	Amino Acid Change	Mutation Type	Mutation Effect	LOH Status
*BRCA1*								
1	22–23	c.5333-?_5467+?del	Unknown	70	p.(Asp1778_His1822del)	Deletion	Frameshift	None
2	18	c.5141_5144del	0.642	80	p.(Val1714fs)	Deletion	Frameshift	*BRCA2* only
3	11	c.3607C>T	0.541	80	p.(Arg1203*)	Substitution	Stop codon	*BRCA2* only
4	15	c.4485-10_4491del	0.362	80	p.(Arg1495fs)	Deletion	Frameshift	None
5	19	c.5154G>A	0.050	90	p.(Trp1718*)	Substitution	Stop codon	*BRCA1* and *BRCA2*
6	11	c.1387_1390delinsGAAAG	0.855	80	p.(Lys463Glufs*17)	Indel	Frameshift	*BRCA1* and *BRCA2*
7	11	c.2263G>T	0.399	60	p.(Glu755*)	Substitution	Stop codon	*BRCA1* and *BRCA2*
8	11	c.3476del	0.704	80	p.(Ile1159fs)	Deletion	Frameshift	*BRCA1* and *BRCA2*
9	5	c.212+3A>G	0.802	70	p.?	Substitution	Splicing	*BRCA1* and *BRCA2*
10	11	c.1961del	0.631	80	p.(Lys654fs)	Deletion	Frameshift	*BRCA2* only
11	24	c.5497G>A	0.927	80	p.(Val1833Met)	Substitution	Missense	*BRCA1* and *BRCA2*
12	11	c.2365del	0.642	70	p.(Ser789Alafs*3)	Deletion	Frameshift	*BRCA2* only
13	11	c.2827A>T	0.304	80	p.(Lys943*)	Substitution	Stop codon	*BRCA1* only
14	11	c.3225_3226del	0.685	80	p.(Asn1075fs)	Deletion	Frameshift	*BRCA1* and *BRCA2*
15	11	c.2188G>T	0.800	80	p.(Glu730*)	Substitution	Stop codon	*BRCA1* and *BRCA2*
16	2	c.3G>A	0.632	70	p.(Met1?)	Substitution	Missense	None
17	16	c.4689C>G	0.802	60	p.(Tyr1563*)	Substitution	Stop codon	None
18	14	c.4484G>T	0.686	40	p.(Arg1495Met)	Substitution	Splicing	*BRCA1* only
19	24	c.5468-2A>G	0.503	20	p.?	Substitution	Splicing	*BRCA2* only
20	11	c.2269del	0.173	10	p.(Val757fs)	Deletion	Frameshift	None
21	11	c.3481_3491del	0.585	70	p.(Glu1161fs)	Deletion	Frameshift	*BRCA2* only
22	21	c.5324T>G	0.628	80	p.(Met1775Arg)	Substitution	Missense	*BRCA1* and *BRCA2*
23	14	c.4372C>T	0.154	70	p.(Gln1458*)	Substitution	Stop codon	None
24	9	c.593+1G>A	0.051	80	p.?	Substitution	Splicing	*BRCA1* only
25	11	c.1603G>T	0.715	80	p.(Gly535*)	Substitution	Stop codon	*BRCA1* only
26	11	c.1390_1391insG	0.674	80	p.(Thr464Serfs*16)	Insertion	Frameshift	*BRCA1* only
27	11	c.1504_1508del	0.743	80	p.(Leu502Alafs*2)	Deletion	Frameshift	*BRCA1* and *BRCA2*
28	16	c.4689C>G	0.818	70	p.(Tyr1563*)	Substitution	Stop codon	*BRCA1* and *BRCA2*
29	19	c.5193+1G>T	0.614	90	p.?	Substitution	Splicing	*BRCA1* only
30	21	c.5296del	0.391	90	p.(Ile1766Serfs*27)	Deletion	Frameshift	*BRCA1* only
*BRCA2*								
31	11	c.4321G>T	0.616	60	p.(Glu1441*)	Substitution	Stop codon	*BRCA2* only
32	11	c.5238dupT	0.681	50	p.(Asn1747fs)	Duplication	Frameshift	*BRCA2* only
33	11	c.5073dup	0.848	80	p.(Trp1692fs)	Duplication	Frameshift	*BRCA1* and *BRCA2*
34	9	c.755_758del	0.711	80	p.(Asp252fs)	Deletion	Frameshift	*BRCA1* and *BRCA2*
35	23	c.8954-1G>A	0.863	70	p.?	Substitution	Splicing	None
36	9	c.712G>T	0.072	50	p.(Glu238*)	Substitution	Stop codon	None
37	8	c.658_659del	0.495	40	p.(Val220fs)	Deletion	Frameshift	*BRCA1* and *BRCA2*
38	11	c.6808_6836del	0.320	40	p.(Gly2270fs)	Deletion	Frameshift	*BRCA2* only
39	21	c.8732del	0.097	80	p.(Ala2911Glufs*16)	Deletion	Frameshift	*BRCA1* only
40	11	c.3339del	0.819	80	p.(Glu1113Aspfs*6)	Deletion	Frameshift	*BRCA2* only
41	8	c.632-1G>A	0.129	80	p.?	Substitution	Splicing	*BRCA1* only
42	11	c.6267_6269delinsC	0.848	40	p.(His2090fs)	Indel	Frameshift	*BRCA1* and *BRCA2*
43	11	c.4631del	0.869	80	p.(Asn1544fs)	Deletion	Frameshift	*BRCA2* only
44	11	c.3545_3546delTT	0.813	40	p.(Phe1182fs)	Deletion	Frameshift	None
45	24	c.9154C>T	0.341	80	p.(Arg3052Trp)	Substitution	Missense	*BRCA1* and *BRCA2*
46	10	c.1859_1865del	0.645	90	p.(Phe620*)	Deletion	Frameshift	*BRCA2* only
47	11	c.5351dup	0.293	80	p.(Asn1784Lysfs*3)	Duplication	Frameshift	*BRCA2* only
48	11	c.3187C>T	0.057	20	p.(Gln1063*)	Substitution	Stop codon	None

LOH, loss of heterozygosity

## Supplementary Materials

The following are available online at https://www.mdpi.com/2072-6694/12/11/3468/s1, Figure S1: Validation data for LOH, Table S1: Detailed summary of pathogenic and likely pathogenic BRCA variants identified, Table S2: Summary of variants of unknown significance identified, Table S3: Metrics of an average sequencing run, Table S4: Summary of validation data for LOH, Table S5: Theorectical heterozygous variant fraction by tumor cellularity due to LOH.
