# Supplementary material for: Tumor BRCA Testing in High Grade Serous Carcinoma: Mutation Rates and Optimal Tissue Requirements
Source: Cancers (Basel). 2020 Nov 21;12(11):3468. doi: 10.3390/cancers12113468 (PMC7700467; doi:10.3390/cancers12113468)
Supplement: Supplementary file 1 [file cancers-12-03468-s001.zip › suppl/cancers-939423 supplementary materials for proofreading_final.docx]

Supplementary Materials:

Tumor BRCA Testing in High Grade Serous Carcinoma: Mutation Rates and Optimal Tissue Requirements

Gulisa Turashvili, Conxi Lazaro, Shengjie Ying, George Charames, Andrew Wong, Krista Hamilton, Denise Yee, Evangeline Agro, Martin Chang, Aaron Pollett and Jordan Lerner-Ellis


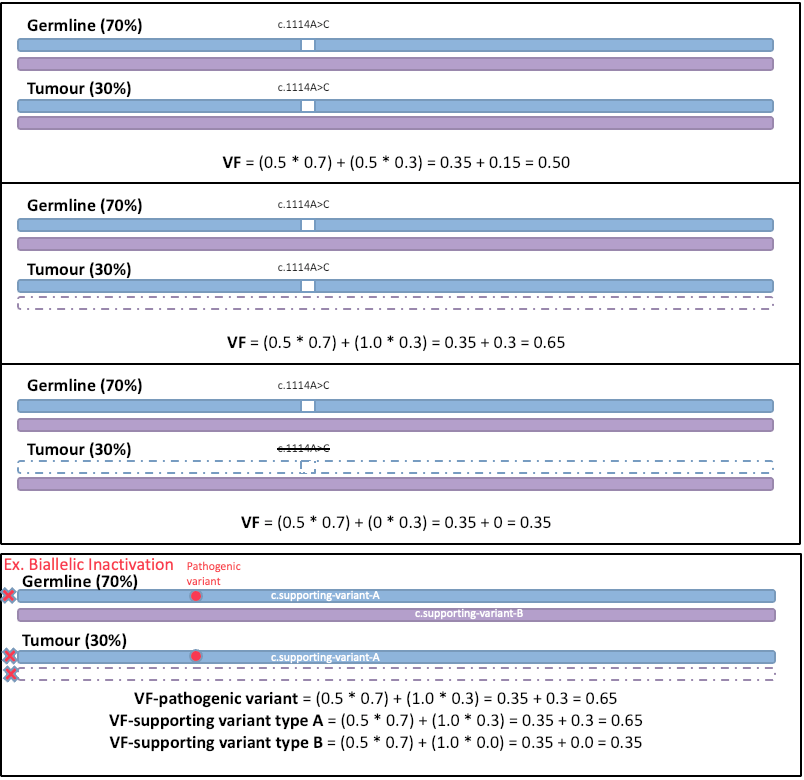


Figure S1: Validation data for LOH

**Table S2.** Summary of variants of unknown significance identified.

| **No.** | **Exon** | **VUS** | **Allele**  **Frequency** | **Amino Acid**  **Change** | **Variant Type** | **Variant Effect** | **LOH Status** |
| --- | --- | --- | --- | --- | --- | --- | --- |
| *BR*C*A1* | | | | | | | |
| 1 | EXON11 | c.1387A>G | 0.67 | p.(Lys463Glu) | Substitution | Missense | *BRCA2* only |
| 2 | EXON11 | c.3784T>C | 0.372 | p.(Ser1262Pro) | Substitution | Missense | None |
| 3 | EXON11 | c.3563G>T | 0.366 | p.(Arg1188Met) | Substitution | Missense | None |
| 4 | EXON02 | c.74C>T | 0.64 | p.(Pro25Leu) | Substitution | Missense | *BRCA1* only |
| 5 | EXON15 | c.4485-8C>T | 0.548 | p.? | Substitution | Splicing | *BRCA1* & *BRCA2* |
| 6 | EXON11 | c.2597G>A | 0.942 | p.(Arg866His) | Substitution | Missense | *BRCA1* only |
| 7 | EXON11 | c.1596A>T | 0.347 | p.(Ile532Ile) | Substitution | Silent | *BRCA1* & *BRCA2* |
| 8 | EXON11 | c.693G>A | 0.957 | p.(Thr231Thr) | Substitution | Silent | *BRCA1* only |
| 9 | EXON11 | c.3635C>T | 0.253 | p.(Ser1212Leu) | Substitution | Missense | *BRCA1* & *BRCA2* |
| 10 | EXON22 | c.5396C>T | 0.06 | p.(Thr1799Ile) | Substitution | Missense | *BRCA1* & *BRCA2* |
| *BRCA2* | | | | | | | |
| 11 | EXON11 | c.3578C>T | 0.21 | p.(Ala1193Val) | Substitution | Missense | *BRCA1* & *BRCA2* |
| 12 | EXON11 | c.3596A>G | 0.33 | p.(Asp1199Gly) | Substitution | Missense | *BRCA1* & *BRCA2* |
| 13 | EXON19 | c.8351G>A | 0.03 | p.(Arg2784Gln) | Substitution | Missense | *BRCA2* only |
| 14 | EXON10 | c.908C>T | 0.06 | p.(Ser303Phe) | Substitution | Missense | None |
| 15 | EXON04 | c.364A>G | 0.496 | p.(Thr122Ala) | Substitution | Missense | *BRCA1* & *BRCA2* |
| 16 | EXON27 | c.9941A>G | 0.38 | p.(Lys3314Arg) | Substitution | Missense | *BRCA2* only |
| 17 | EXON18 | c.8051A>G | 0.674 | p.(Lys2684Arg) | Substitution | Missense | *BRCA1* & *BRCA2* |
| 18 | EXON11 | c.4633_  4635del | 0.477 | p.(Leu1545del) | Deletion | Inframe deletion | *BRCA1* & *BRCA2* |
| 19 | EXON11 | c.5683G>A | 0.053 | p.(Glu1895Lys) | Substitution | Missense | *BRCA1* only |
| 20 | EXON11 | c.5278T>G | 0.607 | p.(Ser1760Ala) | Substitution | Missense | *BRCA1* only |
| 21 | EXON10 | c.1462A>C | 0.311 | p.(Ile488Leu) | Substitution | Missense | *BRCA1* only |
| 22 | EXON10 | c.1462A>C | 0.125 | p.(Ile488Leu) | Substitution | Missense | *BRCA1* & *BRCA2* |
| 23 | EXON25 | c.9501+3A  >T | 0.46 | p.? | Substitution | Splicing | *BRCA1* & *BRCA2* |
| 24 | EXON22 | c.8897T>C | 0.79 | p.(Val2966Ala) | Substitution | Missense | *BRCA2* only |
| 25 | EXON11 | c.3076A>G | 0.17 | p.(Ile1026Val) | Substitution | Missense | *BRCA2* only |
| 26 | EXON17 | c.7868A>G | 0.76 | p.(His2623Arg) | Substitution | Missense | *BRCA1* & *BRCA2* |
| 27 | EXON11 | c.3224G>A | 0.05 | p.(Ser1075Asn) | Substitution | Missense | *BRCA2* only |
| 28 | EXON11 | c.5514G>A | 0.06 | p.(Glu1838Glu) | Substitution | Silent | *BRCA2* only |
| 29 | EXON18 | c.8045C>T | 0.05 | p.(Ala2682Val) | Substitution | Missense | *BRCA1* & *BRCA2* |
| 30 | EXON11 | c.4785G>T | 0.17 | p.(Gln1595His) | Substitution | Missense | *BRCA2* only |
| 31 | EXON11 | c.4620C>T | 0.18 | p.(Asp1540Asp) | Substitution | Missense | *BRCA2* only |
| 32 | EXON02 | c.-10G>A | 0.15 | p.? | Substitution | 5' UTR | *BRCA2* only |
| 33 | EXON09 | c.742G>A | 0.16 | p.(Ala248Thr) | Substitution | Missense | *BRCA2* only |

LOH, loss of heterozygosity; UTR, untranslated region; VUS, variant of unknown significance.

**Table S3.** Metrics of an average sequencing run.

| **Metric** | **Average** | **Average Deviation** | **Upper Limit** | **Lower Limit** |
| --- | --- | --- | --- | --- |
| Cluster Density (K/mm^2^) | 1153 | 89.85 | 1342 | 979 |
| Clusters passing filter (%) | 91.9 | 2.78 | 95.8 | 85.3 |
| Yield (G) | 1.81 | 0.1 | 2.03 | 1.59 |
| ≥Q30 (%) | 94.4 | 0.80 | 95.6 | 92.8 |
| Reads passing filter (M) | 5.75 | 0.32 | 6.45 | 5.03 |
| Percent on-target aligned reads | 94.4 | 1.79 | 97.06 | 83.85 |
| Total aligned reads | 886621.7 | 163445.96 | 1830828 | 213269 |
| Percent Q30 bases | 96.9 | 0.91 | 98.31 | 94.12 |
| Amplicon mean coverage | 3170.3 | 612.33 | 6654.4 | 747.3 |
| Uniformity of coverage  (Pct > 0.2*mean) | 99.6 | 0.58 | 100 | 90.94 |

**Table S4.** Summary of validation data for LOH.

| **Gene** | **HGVS** | **Exon** | **VF in normal tissue** | **VF in Tumor tissue** |
| --- | --- | --- | --- | --- |
| Sample 1 (tumor cellularity 80%) | | | | |
| BRCA2 | c.1114A>C | 10 | 0.481 | 0.653 |
| BRCA2 | c.3396A>G | 11 | 0.513 | 0.322 |
| BRCA2 | c.4563A>G | 11 | 0.998 | 0.998 |
| BRCA2 | c.6513G>C | 11 | 0.999 | 1 |
| BRCA2 | c.6684A>T | 11 | 0.036 | 0.031 |
| BRCA2 | c.7008-2A>T | 13 | 0.506 | 0.648 |
| BRCA2 | c.7242A>G | 14 | 0.512 | 0.337 |
| BRCA2 | c.7397T>C | 14 | 0.998 | 0.992 |
| BRCA2 | c.7806-14T>C | 16 | 0.517 | 0.325 |
| BRCA2 | c.9257-16T>C | 24 | 0.475 | 0.695 |
| BRCA2 | c.9976A>T | 27 | 0.498 | 0.693 |
| BRCA2 | c.10057T>C | 27 | 0.019 | 0.019 |
| BRCA2 | c.10191C>T | 27 | 0.059 | 0.064 |
| BRCA1 | c.4837A>G | 15 | 0.496 | 0.495 |
| BRCA1 | c.4308T>C | 12 | 0.487 | 0.487 |
| BRCA1 | c.3548A>G | 10 | 0.498 | 0.503 |
| BRCA1 | c.3113A>G | 10 | 0.481 | 0.477 |
| BRCA1 | c.2612C>T | 10 | 0.481 | 0.485 |
| BRCA1 | c.2311T>C | 10 | 0.511 | 0.508 |
| BRCA1 | c.2082C>T | 10 | 0.495 | 0.504 |
| BRCA1 | c.1067A>G | 10 | 0.486 | 0.482 |
| Sample 2 (tumor cellularity 20%) | | | | |
| BRCA2 | c.1114A>C | 10 | 0.478 | 0.508 |
| BRCA2 | c.3396A>G | 11 | 0.524 | 0.509 |
| BRCA2 | c.4563A>G | 11 | 0.999 | 0.997 |
| BRCA2 | c.6513G>C | 11 | 0.999 | 0.992 |
| BRCA2 | c.6684A>T | 11 | 0.037 | 0.027 |
| BRCA2 | c.7242A>G | 14 | 0.448 | 0.509 |
| BRCA2 | c.7397T>C | 14 | 1 | 0.997 |
| BRCA2 | c.7806-14T>C | 16 | 0.423 | 0.497 |
| BRCA2 | c.10191C>T | 27 | 0.061 | 0.053 |
| BRCA1 | c.4837A>G | 15 | 0.505 | 0.672 |
| BRCA1 | c.4308T>C | 12 | 0.501 | 0.655 |
| BRCA1 | c.3548A>G | 10 | 0.468 | 0.642 |
| BRCA1 | c.3113A>G | 10 | 0.509 | 0.667 |
| BRCA1 | c.2612C>T | 10 | 0.502 | 0.66 |
| BRCA1 | c.2311T>C | 10 | 0.523 | 0.666 |
| BRCA1 | c.2082C>T | 10 | 0.488 | 0.662 |

LOH, loss of heterozygosity; VF, variant fraction.

**Table S5.** Theoretical heterozygous VF by tumor cellularity due to LOH.

| **Tumor cellularity, %** | **Estimated VF** | |
| --- | --- | --- |
| 90 | 0.05 | 0.95 |
| 80 | 0.1 | 0.9 |
| 70 | 0.15 | 0.85 |
| 60 | 0.2 | 0.8 |
| 50 | 0.25 | 0.75 |
| 40 | 0.3 | 0.7 |
| 30 | 0.35 | 0.65 |
| 20 | 0.4 | 0.6 |
| 10 | 0.45 | 0.55 |

LOH, loss of heterozygosity; VF, variant fraction.

| 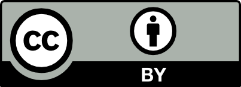 | © 2020 by the authors. Licensee MDPI, Basel, Switzerland. This article is an open access article distributed under the terms and conditions of the Creative Commons Attribution (CC BY) license (http://creativecommons.org/licenses/by/4.0/). |
| --- | --- |
